# Stokes–Einstein Relation in Different Models of Water

**DOI:** 10.3390/molecules29235587

**Published:** 2024-11-26

**Authors:** Sergey Khrapak, Alexey Khrapak

**Affiliations:** Joint Institute for High Temperatures, Russian Academy of Sciences, 125412 Moscow, Russia; khrapak@mail.ru

**Keywords:** transport properties of water, Stokes–Einstein relation, diffusion, viscosity

## Abstract

The purpose of this paper is to discuss to which extent a microscopic version of the Stokes–Einstein (SE) relation without the hydrodynamic radius applies to liquid water. We demonstrate that the self-diffusion and shear viscosity data for five popular water models, recently reported by Ando [J. Chem. Phys. 159, 101102 (2023)], are in excellent agreement with the SE relation. The agreement with experimental results is also quite impressive. The limitations on the applicability of the SE relation are briefly discussed.

## 1. Introduction

Consider a macroscopic sphere of radius *R* immersed in a viscous fluid with dynamic viscosity η and moving with a constant velocity *u* due to the action of an external force. The friction force acting on a moving sphere is given by the well-known Stokes’s formula [1]

(1) F=6πηRu.

This defines the mobility of the sphere $\mu =u/F={\left(6\pi \eta R\right)}^{-1}$. The diffusion coefficient of such a sphere can be obtained from the Einstein relation (also derived independently by Sutherland and Smoluchowski about the same time) $D=\mu k_{B}T$, where $k_{B}$ is the Boltzmann’s constant. This leads us to the conventional macroscopic Stokes–Einstein (or Stokes–Einstein–Sutherland–Smoluchowski) relation [2]:

(2) $$D=\frac{k_{B}T}{6\pi \eta R}.$$

The numerical coefficient in Equation (1) corresponds to the “stick” boundary condition at the sphere surface. The question arises regarding how the Stokes–Einstein (SE) relation should be modified when the radius of the sphere decreases and becomes comparable to the characteristic distance of intermolecular interactions. In this situation, it is common to replace the actual size of the sphere with the so-called *hydrodynamic radius* $R_{H}$, which is often considered as an adjustable parameter. Further to the microscale, when self-diffusion of atoms in simple pure fluids is considered, the SE relation becomes

(3) $$D\eta \left(\frac{\Delta }{k_{B}T}\right)=\alpha_{\mathrm{SE}},$$

where $\Delta =\rho^{-1/3}$ is the mean interatomic separation and ρ is the atomic number density. Comparing Equations (2) and (3), we observe that the role of the effective tracer sphere radius is now played by the characteristic interatomic separation Δ. Equation (3) is sometimes referred to as the SE relation without the hydrodynamic radius [3]. The numerical coefficient $\alpha_{\mathrm{SE}}$ is only weakly system and state-dependent, as discussed in more detail below.

This form of the SE relation at microscales was discussed by Frenkel [4] using qualitative arguments. More convincing quantitative models have been developed later [5,6]. In particular, the model developed by Zwanzig [5] is based on a vibrational picture of atomic dynamics [7] and allows to derive an explicit relation for $\alpha_{\mathrm{SE}}$. Based on the Debye approximation for the collective excitation spectrum, the SE constant can be expressed in terms of the instantaneous longitudinal ($c_{l}$) and transverse ($c_{t}$) sound velocities. The resulting expression is $\alpha_{\mathrm{SE}}≃0.132\left(1+c_{t}^{2}/2c_{l}^{2}\right)$ [8]. Taking into account limitations on the ratio $c_{t}/c_{l}$, it is possible to confine the SE coefficient in a relatively narrow range $0.132≲\alpha_{\mathrm{SE}}≲0.181$ [5]. The SE coefficient depends, though weakly, on the ratio $c_{t}/c_{l}$ and hence on the softness of interatomic interaction. In plasma-related fluids characterized by very soft Coulomb-like interactions, the inequality $c_{t}\ll c_{l}$ holds, and $\alpha_{\mathrm{SE}}$ tends to its lower limit [8,9]. In the hard-sphere limit, the transverse-to-longitudinal velocity ratio approaches $c_{t}/c_{l}∼0.5$ [10] and $\alpha_{\mathrm{SE}}$ increases toward the upper limit [9]. Importantly, no concepts of hydrodynamic radius or boundary condition are required in this microscopic formulation of the SE relation.

The applicability of the microscopic SE relation without the hydrodynamic radius to dense simple fluids has been numerously confirmed for various systems. These include simple models such as Coulomb (one-component plasma) and screened Coulomb (complex or dusty plasma) fluids of charged particles [11,12,13], soft (inverse power law) repulsive particle fluid [14], Lennard–Jones fluid [3,15,16], Weeks–Chandler–Andersen fluid [17,18], and the hard sphere fluid [9,17,19]. Among real liquids, in which SE relation without the hydrodynamic radius is satisfied, let us mention liquid iron at conditions of planetary cores [20], dense supercritical methane (at least for the most state points investigated) [21,22], and silicon melt at high temperatures [23]. The Stokes–Einstein relation is also satisfied, with some reservations, in valence-limited disordered fluids of patchy particles [24]. Several important non-spherical molecular liquids have been examined using numerical simulations, and the applicability of the SE relation has been confirmed for some linear (N${\,}_{2}$, O${\,}_{2}$, F${\,}_{2}$, Cl${\,}_{2}$, CO${\,}_{2}$, and CS${\,}_{2}$), chain (n-alkanes up to C${\,}_{10}$H${\,}_{22}$), and discotic (C${\,}_{6}$H${\,}_{6}$ and C${\,}_{10}$H${\,}_{8}$) molecules [25]. It has been also reported that water, modeled with the TIP4P/2005 model [26], is consistent with the SE relation without the hydrodynamic radius [25].

Since water is arguably the most ubiquitous and important liquid relevant for our life, it is important to know whether the SE relation is satisfied and under which conditions. Water properties are of great practical importance, but they are far from being fully understood, especially taking into account a number of unusual anomalies exhibited by water in comparison to conventional simple fluids [27]. Not surprisingly, a large number of water models have been developed to reproduce its various properties in molecular simulations. There have been indications that the SE relation is indeed satisfied in various water models, even though discrepancies in transport coefficients between different models can be significant. It has been recently demonstrated [28] that the results of Yeh and Hummer [29] using the TIP3P model and recent “magic box” results by Busch and Paschek [30] using the TIP4P/2005 water model are consistent with the SE relation of Equation (3). Recently, extensive simulations of the water transport coefficients in a wide range of thermodynamic conditions using the TIP4P/Ice model, which was specially designed to cope with water near the fluid–solid phase transition and solid-phase properties [31], have been reported [32]. These simulation results agree with the SE relation without the hydrodynamic radius [33].

## 2. Materials and Methods

Let us first remind the physical picture behind the SE relation in dense simple fluids emerging from the vibrational picture of atomic dynamics. In this case, atomic diffusion can be described as a random walk process [13]. The time scale of this process is the Maxwell relaxation time

(4) $$\tau_{M}=\frac{\eta }{G_{\infty }},$$

where η is the coefficient of shear viscosity and $G_{\infty }$ is the instantaneous (infinite frequency) shear modulus (see Ref. [34] for recent examples of calculating $\tau_{M}$). This can be considered as a time during which an atom local equilibrium position remains fixed before jumping to a new equilibrium position corresponding to a new local minimum of the multidimensional potential energy surface. In this approximation, the consecutive equilibrium positions exhibit a random walk, and this is why fluids can flow, in contrast to crystalline solids. On the other hand, atoms in dense fluids exhibit solid-like vibrations around these local equilibrium positions. The corresponding amplitude is

(5) $$\langle \delta r^{2}\rangle =\frac{6k_{B}T}{m}\langle \frac{1}{\omega^{2}}\rangle ,$$

where *m* is the atomic mass, ω is the vibrational frequency, and the averaging should be performed considering only sufficiently high frequencies $\omega \tau_{M}>1$. The coefficient of two appears, because the initial atom position (after a jump of the local equilibrium position) as well as the final one (just before a new jump) are randomly distributed around the current local equilibrium position. Moreover, this vibrational amplitude appears considerably larger than the distance between consecutive equilibrium positions. This may not be the case in the super-cooled regime [35,36,37], but this is irrelevant for the present consideration. Therefore, it is the vibrational amplitude that defines the characteristic length scale of the random walk process. The diffusion coefficient is then

(6) $$D=\frac{k_{B}T}{m\tau_{M}}\langle \frac{1}{\omega^{2}}\rangle .$$

Zwanzig’s result is recovered using the Debye approximation for the collective excitation spectrum, as discussed in detail in Ref. [13].

Now, as we pointed out in the Introduction, the SE relation without the hydrodynamic radius operates not only in simple atomic fluids but also in molecular fluids like water. An important question, whether the SE relation is valid generally or only for particular models of water, remains to be answered. New insight is now available thanks to extensive numerical simulations reported by Ando [38]. This study reports a detailed calculation of the shear viscosity for the four-point Optimal Point Charge (OPC) and three-point OPC (OPC3) water models. The OPC water model was proposed in Ref. [39] and reported to reproduce a comprehensive set of bulk properties significantly more accurately than other commonly used rigid models. In the approach, no geometry constraints other than the symmetry are imposed, and the distribution of point charges is optimized to best describe the “electrostatics” of the water molecule [39]. This approach was further applied to develop a computationally effective OPC3 water model, which was shown to be significantly more accurate than the commonly used water models of same class [40].

Ando has calculated the shear viscosity coefficients of the OPC and OPC3 water models in the temperature range from 273 to 373 K using the Green–Kubo formalism. In addition, other important properties, such as the density, self-diffusion coefficient, surface tension, and static dielectric constant are evaluated. Moreover, the same properties are calculated for the TIP4P/2005, TIP4P-FB, and TIP3P-FB [41] water models for comparison. It turns out that the OPC and OPC3 models yield similar values of the shear viscosity over the examined temperature range. The calculated shear viscosities at higher temperatures compare well with the experimental results. However, a deviation from the experimental values is pronounced at temperatures below 313 K. For example, at 298 K and 273 K, the OPC and OPC3 models predict the shear viscosities that are 10% and 20% lower than the experimental values, respectively. The shear viscosities predicted using the OPC and OPC3 models are lower than those based on TIP4P/2005, TIP4P-FB, and TIP3P-FB but only for temperatures below 293 K. The recommended experimental references values for the self-diffusion coefficient from Ref. [42] and the recommended values for the density and viscosity from the NIST database [43,44,45,46] are also summarized. This provides us with the unprecedented dataset of water properties corresponding to various models and experiments. We use this excellent opportunity to calculate and verify the validity of the SE relation in water.

## 3. Results

**Figure 1:**
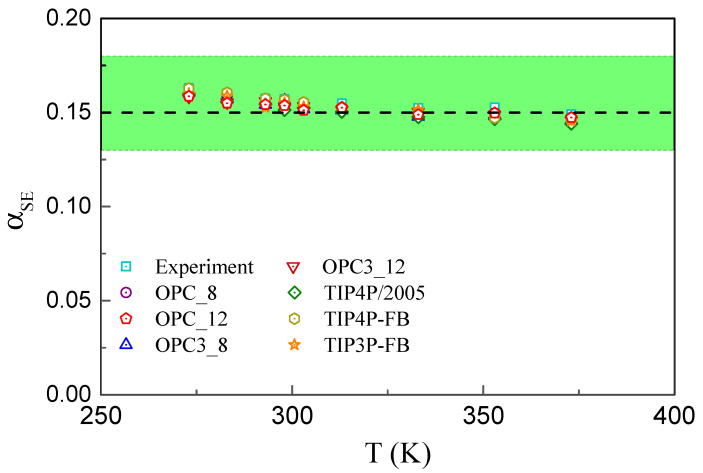
(Color online) Stokes–Einstein relation in water: The SE product $D\eta \left(\Delta /k_{B}T\right)=\alpha_{\mathrm{SE}}$ as a function of temperature *T*. Shaded (green) region corresponds to the theoretically expected range, $0.13≲\alpha_{\mathrm{SE}}≲0.18$. Symbols are calculated from the extensive database provided by Ando in Ref. [38]. They represent different water models and their implementation; see the legend and the text. The SE coefficients are close to a constant value ≃0.15, as shown by the dashed line.

The main results are shown in Figure 1, where the dependence of the SE coefficient $\alpha_{\mathrm{SE}}$ on the temperature is plotted. For the OPC and OPC3 models, two different cutoff distances, 8 and 12 Å are used, and this is reflected in the figure legend. Nine different temperatures, 273, 283, 293, 303, 313, 333, 353, and 373 K are examined. Other details regarding the simulation protocol and methods employed can be found in Ref. [38]. The symbols in Figure 1 correspond to the results from the simulations and experiment. The (green) shaded region marks the theoretical expectation $0.13≲\alpha_{\mathrm{SE}}≲0.18$. All the data points are confined in the expected range. Moreover, all the data points are almost perfectly coinciding for each temperature point. Thus, in the parameter regime investigated, the SE relation without the hydrodynamic radius holds with an excellent accuracy for all the water models considered. Note that the shear viscosity coefficient varies from one model to another, in particular in the low-temperature regime; see Figure 2 from Ref. [38]. Hence, the self-diffusion coefficient is also model-dependent, whilst their product in the form of Equation (3) is not.

One potentially important consequence of our result has been pointed out by one of the anonymous referees. This is the possibility of estimating the Schmidt number, which is a dimensionless number defined as the ratio of momentum diffusivity (kinematic viscosity) and mass diffusivity (diffusion coefficient),

(7) $$\mathrm{Sc}=\frac{\nu }{D}=\frac{\eta }{m\rho D}.$$

By virtue of the SE relation in the form of Equation (3), we obtain

(8) $$\mathrm{Sc}=\frac{\eta^{2}\Delta^{4}}{\alpha_{\mathrm{SE}}mk_{B}T}.$$

This implies that in dense water, the Schmidt number can be estimated without knowing the diffusion coefficient. Dynamic viscosity, density, and temperature are sufficient for this purpose. The turbulent Schmidt number is beyond the scope of this paper.

## 4. Discussion

We can observe in Figure 1 that the SE coefficients slightly but systematically decrease as the temperature increases. The same trend has been documented in previous studies, see, e.g., Figure 5 of Ref. [32]. Ohtori et al. using the TIP4P/2005 model observed that the SE coefficient remains constant close to 1/2π≃0.16 for T≲320 K and then somewhat drops for higher temperatures; see Figure 2 of Ref. [25]. No such drop is observed in the dataset from Ref. [38]. As we discussed in Ref. [33], some decrease in $\alpha_{\mathrm{SE}}$ might be the indication of the potential softening upon the temperature increases. This would lead to lower values of the transverse-to-longitudinal sound velocity ratio and hence to $\alpha_{\mathrm{SE}}$, which is similar to the situation in simple fluids [8]. Alternatively, this decrease can manifest the approach to the super-cooled behavior, where SE relation breaks down. These aspects require further consideration. Apart from a weak decrease, the values of the SE coefficient are close to ≃0.15, which is similar to the case of the Lennard–Jones fluid [9].

It should be noted that outside the parameter regime studied, the violation of the SE relation should not come as a surprise. The SE relation is generally violated in the supercritical fluid region above the Frenkel line on the phase diagram, where the dynamics becomes gas-like [9,47,48,49]. This can be understood as follows: in the liquid-like regime, the viscosity coefficient η decreases with temperature, while the diffusion coefficient *D* increases with *T*. Hence, it is reasonable to expect that their product, Dη, varies little with temperature. In the gas-like regime, both η and *D* increase with temperature. Hence, in this regime, Dη is a strongly increasing function of temperature. This is intimately related to viscosity having a minimum due to the change of the main mechanism of momentum transfer upon gas-like to liquid-like dynamical crossover; see Refs. [50,51].

Another important regime where SE relation is known to fail corresponds to strongly super-cooled liquids approaching the glass transition. In this regime, a fractional version of SE relation is often considered as a more appropriate alternative [52,53,54]. Relevant numerical results exist [35,36,37,55], pointing to an increased relevance of translational jump diffusion in the super-cooled regime and associated diffusion–viscosity decoupling. This important topic is, however, beyond the scope of the present work.

## 5. Conclusions

To summarize, we have verified the validity of the SE relation in water using an extensive dataset published recently by Ando [38]. Five popular water models have been used to calculate density as well as the coefficients of shear viscosity and diffusion. We demonstrate that the microscopic SE relation without the hydrodynamic radius, Equation (3), remains very accurate with $\alpha_{\mathrm{SE}}≃0.15$ for all water models in the temperature range investigated. Remarkably, no free parameters or adjustable coefficients are involved. Interestingly enough, while the self-diffusion and viscosity coefficients are model-dependent, their product in the form of Equation (3) is not. We point out that in the regime considered, the Schmidt number can be estimated without knowing the diffusion coefficient. The limitations on the applicability of the SE relation from the side of low and high temperatures and densities are briefly discussed. Altogether, these results represent an important step toward better understanding the transport properties of water across its phase diagram.

## Data Availability

Data used in this study are available from the corresponding author upon reasonable request.

## Abbreviations

The following abbreviations are used in this manuscript:

SE	Stokes–Einstein
TIP	Transferable Intermolecular Potential
OPC	Optimal Point Charge
